# Retrospective screening for congenital cytomegalovirus infection in the Survey of Neonates in Pomerania shows very low disease burden

**DOI:** 10.1186/s40348-026-00231-6

**Published:** 2026-04-10

**Authors:** Jan Baier, Chiara Konrad, Maximilian Groffmann, Till Ittermann, Heike Allenberg, Maria Asmus, Friedrich Ihler, Chia-Jung Busch, Kathrin Lehmann, Karsten Becker, Anja Lange, Matthias Heckmann

**Affiliations:** 1 Department of Neonatology and Pediatric Intensive Care, University Medicine Greifswald, Greifswald, Germany; 2 DZKJ (German Centre for Child and Adolescent Health), Partner Site Greifswald/Rostock, Greifswald, Germany; 3 Institute of Community Medicine, Division of Health Care Epidemiology and Community, University Medicine Greifswald, Greifswald, Germany; 4 Department of Otorhinolaryngology, Head and Neck Surgery, University Medicine Greifswald, Greifswald, Germany; 5 Friedrich Loeffler-Institute of Medical Microbiology, University Medicine Greifswald, Greifswald, Germany

**Keywords:** Congenital cytomegalovirus, Newborn screening, Prevalence, Disease burden, Sensorineural hearing loss

## Abstract

**Background:**

Congenital cytomegalovirus (cCMV) infection is considered one of the most common infectious pathologies in neonates, showing a wide range of clinical manifestations, ranging from completely asymptomatic to critically ill newborns. Primary infection during the first trimester of pregnancy carries the highest risk of severe fetal involvement. However, the majority of infected fetuses show an asymptomatic or mild course of disease. The most common long-term sequela is sensorineural hearing loss, which is detected in only about 50% of affected children during newborn hearing screenings. Furthermore, epidemiological data on disease burden remain limited and show considerable variability. The aim of this study was to investigate the prevalence of cCMV and its associated disease burden at follow-up, particular sensorineural hearing loss, in a population-based birth cohort.

**Results:**

We conducted a retrospective analysis for cCMV in *n* = 1995 newborns using urine and plasma biosamples from the second baseline cohort of the Survey of Neonates in Pomerania (SNiP-II). Four participants (prevalence rate 0.2%) were tested positive. Two of these cases had already been identified shortly after birth, having been screened for cCMV due to clinical suspicion. The seroprevalence of IgG antibodies among pregnant women was 47.6%. For follow-up, the patient database of our Department of Otolaryngology, Head and Neck Surgery (ENT) was reviewed for hearing pathologies in all children born during the SNiP-II recruitment period (2013–2017). Twelve out of *n* = 1138 SNiP-II participants (1.1%) showed confirmed sensorineural hearing loss (SNHL), none of which were associated with cCMV. A complete clinical follow-up of the cCMV-positive cases revealed no sequelae related to the infection.

**Conclusions:**

By using a population-based retrospective analysis approach, we demonstrated a very low prevalence and disease burden of cCMV in our region. Our data supports the current guideline recommendations of a risk factor-based screening strategy.

## Background

Infections with human cytomegalovirus (CMV) represent a worldwide health burden, and congenital cytomegalovirus (cCMV) is considered the most common infectious cause for embryo- and fetopathies in high-income countries, with an estimated overall prevalence rate at birth of 0.67% [1].

cCMV is associated with several sequelae, of which sensorineural hearing loss (SNHL) and neurodevelopmental disability in childhood are the most significant [2, 3]. The majority of newborns with cCMV are asymptomatic at birth. However, approximately 10% will develop SNHL within the first 5 years of life. In contrast, around 30–50% of the symptomatic newborns already have or will develop SNHL, making it the most important long-term complication of cCMV [4, 5].

As SNHL occurs during the critical period of speech and language development, early detection of affected infants is crucial to support proper development and to reduce the associated societal and economic burden [6–8]. Additionally, there is growing evidence that early medical treatment of cCMV affected children may be beneficial and can improve hearing and developmental outcomes [9, 10].

Nevertheless, in most countries, cCMV screening programs do not exist, and therefore, many newborns with cCMV – even those who are symptomatic – remain undiagnosed due to the often subtle and non-specific symptoms [11]. Importantly, a definitive diagnosis of cCMV in neonates can only be confirmed during the first three weeks of life. Furthermore, currently only two countries, Italy and France, have national recommendations for CMV screening in pregnant or pre-conceptional women, although studies have shown that preventive hygienic measures in women at increased risk of CMV exposure can reduce the rate of primary infections by up to 50% [12–14]. Vertical transmission can be reduced by early valganciclovir treatment. However, as vertical transmission can only be confirmed invasively by amniocentesis, and a positive result does not necessarily indicate fetal disease, there is an ongoing discussion about the indications of a prenatal screening. On the other hand, there is increasing evidence that postnatal screening improves child health outcomes and is cost-effective [15, 16].

Hence, we aimed to provide further data on cCMV disease burden using a population-based approach in the region of Western Pomerania in north-eastern Germany. By linking neonatal data with hearing pathologies, we seek to contribute further evidence to the ongoing discussion on cCMV screening [14].

## Methods

### Study population

The Survey of Neonates in Pomerania (SNiP) is a population-based birth cohort study conducted in Western Pomerania, Germany. It comprises two birth cohorts (SNiP-I and SNiP-II) with both including longitudinal follow-up surveys [17]. The second cohort additionally includes urine biosamples collected at birth. The present analyses were conducted within SNiP-II, with participant enrollments between 2013–2017. This baseline cohort comprises a database with more than 250 variables, along with biosamples from 3502 mother-infant pairs, representing 54% of all livebirths (*n* = 6487) in the study region. A complete dataset was available for *n* = 3435 children (Table 1). Biosamples, including urine, plasma, cord-blood, and placental tissue were collected during or immediately after birth.

**Table 1:** Characteristics of the baseline cohort and subgroups in that study

	SNiP-II total cohort (*n* = 3435)	SNiP-II biosamples (*n* = 1995)	*p*-value	SNiP-II ENT visit (*n* = 1138)	*p*-value
GA (weeks)	39 (36–41)	39 (35–41)	< 0.001	39 (33–41)	< 0.001
birth weight (grams)	3380 (2436–4010)	3340 (2290–4000)	< 0.001	3130 (1930–3980)	< 0.001
birth length (cm)	51 (46–54)	51 (45–54)	< 0.001	51(43–54)	< 0.001
male sex, n (%)	1732 (50.4)	1034 (51.8)	0.052	604 (53.1)	0.029
HC at birth (cm)	35 (32–36.5)	35 (32–36.5)	< 0.001	34 (31–36.5)	< 0.001
preterm birth (< 37 weeks GA), n (%)	454 (13.2)	320 (16.0)	< 0.001	347 (30.5)	< 0.001
LBW, n (%)	382 (11.1)	279 (14.0)	< 0.001	296 (26.0)	< 0.001
VLBW, n (%)	75 (2.18)	52 (2.61)	0.046	61 (5.36)	< 0.001
SGA, n (%)	297 (8.65)	221 (11.1)	< 0.001	234 (20.6)	< 0.001
SGA (< 3rd percentile), n (%)	86 (2.50)	61 (3.06)	0.014	69 (6.06)	< 0.001
microcephaly (HC < 3rd percentile), n (%)	64 (1.86)	52 (2.61)	< 0.001	55 (4.83)	< 0.001

Continuous variables are presented as median (10th–90th percentiles) due to non-normal distributions; group comparisons were performed using the Mann–Whitney U test. Categorical variables are shown as absolute counts (n) and percentage (%) and compared using the chi-squared test

*Abbreviations*: *ENT* Department of Otolaryngology, Head and Neck Surgery, *GA* gestational age, *HC* head circumference, *SGA *small for gestational age, *SNiP* survey of Neonates in Pomerania, *(V)LBW* (very) low birth weight

### Biosample preparation and processing

All biosamples were stored at −80 °C immediately after collection. Detection of CMV-DNA was performed using the AltoStar® CMV PCR Kit 1.5 (altona diagnostics, Hamburg, Germany), according to the manufacturer´s protocol, at the Institute of Medical Microbiology. Briefly, 100–350 µl of plasma or urine were diluted with sample buffer to a final volume of 700 µl per sample, from which, 45 µl of nucleic acid was automatically extracted. For PCR amplification, 10 µl of the obtained eluate were added to the master mix. For each run, 93 samples, two positive controls and one negative control were analyzed on a 96 well plate using the CFX96 Dx Real-Time PCR System (Bio-Rad, Munich, Germany). Data analysis and validation of the results was done using CFX Manager DX Software, according to the manufacturer´s instructions. The assay demonstrates a sensitivity of 99% and a specificity of 100% for urine samples, whereas for plasma samples, sensitivity ranges from 65–90% with a specificity of 100% [18, 19].

### Statistical analyses

Statistical analyses were performed using Stata version 19.5. (Stata corporation, College Station, Texas, USA). All continuous variables were tested for normality using histograms, Q-Q plots, and Shapiro–Wilk tests. As none of the variables followed a normal distribution, continuous variables are reported as medians with 10th–90th percentiles. Comparison of groups was performed using the Mann–Whitney U test. Categorical variables are summarized as counts and percentages and were compared using the chi-squared test.

## Results

### Study design and findings

**Fig. 1:**
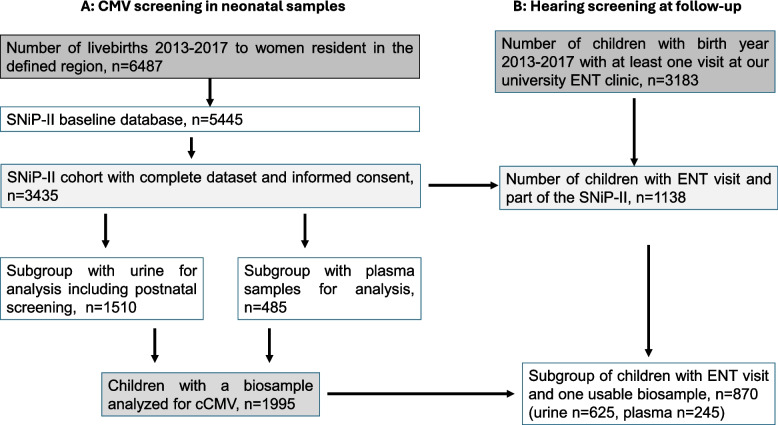
cCMV (congenital cytomegalovirus) screening in neonatal urine and plasma samples in the SNiP-II birth cohort study (**A**) and follow-up in the ENT clinic (**B**). Abbreviations ENTDepartment of Otolaryngology, Head and Neck Surgery, SNiP- survey of Neonates in Pomerania

We conducted a retrospective, multistep analysis to determine cCMV status in the SNiP-II cohort (Fig. 1 A), followed by an analysis of participants who underwent examination for hearing pathologies in the ENT department (Fig. 1B).

All available urine samples from the SNiP-II cohort (*n* = 1642) were screened. After applying several quality criteria, *n* = 1404 samples were considered suitable for CMV-DNA analysis. An additional *n* = 106 newborns of the SNiP-II cohort had already undergone urine cCMV screening after birth due to clinical suspicion (e.g. microcephaly, thrombocytopenia, pathological ultrasound findings). These samples were not reanalyzed; instead, their results were incorporated into the study cohort, yielding a total of *n* = 1510 urine samples.

To further expand the study group and ensure inclusion of the children presented at the ENT clinic, *n* = 485 plasma samples were additionally analyzed.. This included, *n* = 245 plasma samples from children with ENT presentations and *n* = 240 matched plasma samples from the SNiP-II cohort. Matching was performed based on key clinical variables such as gestational age, birth weight, lengths, head circumference, APGAR score, lactate at birth, NICU admission, to minimize selection bias.

In total, *n* = 1995 children from the SNiP-II cohort (30,7% of all live births) were screened for cCMV. Since all available biosamples of participants with ENT presentation were included, the biosample cohort differed slightly from the SNiP-II cohort in baseline characteristics, although these differences were limited (Table 1).

Four children had a positive result for CMV-DNA, defining a prevalence of 0.2%. Two of these cases (twins) were already tested positive directly at birth, while two additional cases were identified retrospectively through urine analysis. The mother of the twins was tested positive for CMV IgG and negative for IgM at delivery; no prenatal CMV testing was performed. The mothers of the other two positively tested newborns were not tested for CMV at all. The twins were symmetrically small for gestational age (SGA) and showed periventricular vasculopathy on cranial ultrasound. Therefore, they were treated with valganciclovir for six months. Among children evaluated in the ENT clinic, *n* = 870 of 1138 had available biosamples and underwent CMV screening. Additionally, *n* = 11 out of 12 with children with confirmed SNHL were tested, but none of these children were tested positive for cCMV.

Based on the analysis of *n* = 1510 urine samples and correction for test quality, we estimate that 17.36 children (95%-confidence interval (CI) of 5 to 44 cases) were born with cCMV in our region between 2013–2017. Assuming that approximately 24% of infected children develop symptoms at birth or during infancy, this would correspond to an estimated 4.17 symptomatic cases (95% CI 1.14–10.64) [20, 21].

For follow-up, all SNiP-II participants we screened for possible SNHL. By matching the SNiP-II database with ENT department records of all children born between 2013 and 2017, we identified *n* = 1138 SNiP-II participants (33.1% of the cohort) with at least one outpatient or inpatient ENT presentation. This ENT subgroup included 76.4% of all preterm infants in SNiP-II and therefore showed differences in the baseline perinatal characteristics (Table 1). A total of *n* = 58 patient files could be identified with an ICD-10 diagnosis H90.3–90.8 *(*codes for sensorineural hearing loss)*.* All these cases were reviewed in detail, regarding clinical reports (doctor´s letter), diagnostic testing (physical and device-related examinations) and surgical documentation. Ultimately, SNHL was confirmed in *n* = 12 children.

The median age at diagnosis for the confirmed SNHL cases was 3 years and 26 days, with the youngest child receiving SNHL diagnosis being only nine days old, to the oldest, 6 years, 9 months and 18 days. Seven children suffered from bilateral hearing loss and 5 had monolateral involvement. Three children with bilateral hearing loss received bilateral cochlea implants, while the remaining 9 received hearing aids. Ten of the twelve children were diagnosed with delayed speech and language development. All SNiP-II participants with a ENT record and available biosamples (*n* = 870), were retrospectively screened for cCMV.

All children who were tested positive for cCMV infection were invited for detailed follow-up assessment. One family could not be contacted, and another family could not manage to attend the follow-up but reported normal development and no hearing impairment during a telephone interview. The family of the treated twins, who had remained asymptomatic during regular checkups until the age of five, received a complete examination. This comprehensive examination included: 1.) physical examination, 2.) extended questionnaire on behavior, neurophysiological development, social interactions [22–26], 3.) an audiological examination in our ENT clinic and 4.) a developmental test by our trained specialists using the Bruininks-Oseretsky assessment (BOT-2) [27]. This resulted in no pathological findings that could be related to cCMV.

### Additional analyses

We analyzed CMV serological screening data from women who gave birth at our hospital between 2013 and 2017 (total number of births *n* = 4826), corresponding to the time when the SNiP-II baseline recruitment was conducted. Among *n* = 1633 women who were screened for CMV-antibodies, *n* = 777 were serum IgG positive (seroprevalence 47.6%).

In addition, we also evaluated the clinical indications for postnatal cCMV screening in *n* = 106 newborns, based on the indications provided by the attending physician. The most common indications were prematurity (*n* = 58), perinatal distress or failure to adapt (*n* = 46), small for gestational age (*n* = 30), hyperbilirubinemia requiring treatment (*n* = 28), severe hypoglycemia (*n* = 25), neurological abnormalities (*n* = 25), and suspected perinatal infection (*n* = 19). Multiple indications were possible for each patient.

Finally, our clinical patient records were screened for confirmed cCMV diagnoses. Apart from the two siblings identified within the SNiP-II cohort, no additional cases were found among study participants born in the region between 2013–2017. However, one newborn with symptomatic cCMV was identified, who was born within the recruitment area during the study period but was not enrolled in the SNiP-II study. The child was diagnosed with sensorineural hearing loss of the left ear and delayed motor development during follow-up.

## Discussion

We conducted a retrospective analysis of cCMV infection in a birth cohort spanning five years of livebirths in the region of Western Pomerania. By analyzing *n* = 1995 biosamples and checking *n* = 1138 patient records from the ENT department, we identified *n* = 4 children with a positive CMV-DNA result, none of whom developed sequelae of cCMV. The overall detection rate of CMV-DNA in our study was 0.2%, which corresponds to the lower range of recently reported prevalence rates in high-income countries [20, 28, 29]. Recent large-scale studies using dried blood spot (DBS) samples from newborn screening programs have reported prevalence rates between 0.11% and 0.5% [21, 30]. Since sensitivity of DBS is considered inferior to urine samples, the true prevalence in those cohorts is likely underestimated [19]. To our knowledge, this study is the first to use urine samples as the main source to test for cCMV. Given that urine sampling provides superior diagnostic sensitivity for CMV detection in neonates, the detection rate observed in our study likely reflects the true regional prevalence. The seroprevalence of CMV IgG antibodies among women giving birth during the study period was 47%, consistent with the previous reports from Germany [20, 31]. Taken together, our findings are consistent with the existing data on cCMV epidemiology in our country, which is nevertheless still very limited.

Several factors may explain why no additional symptomatic cCMV cases were identified in our study group. Firstly, due to the lack of a generalized screening for cCMV in our country, epidemiological data on cCMV prevalence is still scarce and heterogeneous. Our initial power analysis assumed a prevalence of 0.5% cCMV in high-income countries [32], with 24% of positive cases being symptomatic at birth or developing sequelae later in childhood [20, 21]. Based on these assumptions, a minimum sample size of *n* = 1918 would be required to detect a symptomatic child with 90% power. However, given the lower prevalence (0.2%) observed in our cohort, the power dropped to 61.7% and a substantially larger sample size (*n* = 4796) would have been necessary to achieve 90% power. Secondly, we may have missed SNiP-II participants with cCMV, since we analyzed only 57% of the SNiP-II cohort and notably, *n* = 485 of the1995 biosamples were plasma samples, as urine was not available for these participants. Although detection of CMV-DNA seems to be more sensitive in plasma than in DBS samples [18, 33], this diagnostic approach is considered to be of inferior sensitivity in comparison to urine samples because of low viremia levels in newborns [19]. Overall, we could screen 30.7% of all livebirths between 2013 and 2017 in our region and identify four positive cases.

In order to reduce potential underdetection of clinically relevant outcomes, we additionally reviewed all ENT records of SNiP-II participants up to August 2023. Since our institution represents the only clinical center for diagnostics of hearing loss in childhood in our rural region, it is reasonable to assumed that most children with suspected hearing problems are referred to our University Clinic. At the time of follow-up, the youngest children in the cohort were 5–6 years old, which falls within the critical period during which cCMV-associated sensorineural hearing loss typically manifests and should be monitored regularly [4].

Certainly, some families have moved away from our region after the child´s birth and therefore might have escaped our ENT follow-up approach to screen for cCMV sequelae. Among *n* = 1138 children evaluated in the ENT department, 12 were diagnosed with confirmed SNHL and 11 of them tested negative for cCMV. The remaining child who was not tested for cCMV was diagnosed with Usher syndrome. The twins who were tested positive for cCMV at birth, remained asymptomatic throughout follow-up, despite receiving antiviral therapy. This case shows the clinical uncertainty associated with managing newborns who test positive for CMV-DNA but present with minimal or no clinical signs. Following parental consent, antiviral treatment was initiated and to date both children have not shown any cCMV related sequelae. Additionally, one newborn that was born in 2014, but not enrolled in SNiP-II was diagnosed with cCMV at our hospital and received antiviral treatment. This child later developed monolateral hearing loss and developmental delay.

Overall, based on our study data, we can summarize that our region has a very low disease burden associated with cCMV. Although, there is no general cCMV screening in our region, there is general awareness of the disease, and pregnant women are generally informed early on about the risk of infection, particularly when in contact with young children. Therefore, from our point of view, it remains debatable, whether a general cCMV screening would be both clinically benefitting and cost-effective. Especially in a low prevalence setting like ours, the number needed to test is very high, approximately *n* = 500 to detect one positive case and *n* = 2000 to identify one symptomatic case.

While the quality of DBS testing for cCMV has improved recently, it still lacks sensitivity and is prone to miss at least one in ten positive newborns. Transitioning to universal urine-based screening might pose practical challenges of applying urine collection bags to newborns, which regularly detach and may disrupt the critical early period of parent-infant bonding. Even if such a strategy was implemented successfully, identifying additional asymptomatic cases would introduce further clinical and ethical dilemmas. Most CMV-positive newborns remain asymptomatic during childhood, and physicians would encounter highly delicate situations in maternity wards presenting a positive test result but no general solution, as reliable prognostic markers for disease progression are still lacking. There is some evidence suggesting that an increased viral load in blood, but not in saliva or urine, is associated with development of symptoms in early childhood and that a “spontaneous early viral suppression” seems to be an indicator for asymptomatic courses [34–36]. Additionally, studies with small sample sizes indicate that failure in newborn hearing screening and cerebral abnormalities in neuroimaging after birth are risk factors for later sequelae [35, 37]. Importantly, the treatment with antiviral substances has shown that it does not reliably prevent development of later symptoms in initially asymptomatic newborns [38]. In contrast, RCTs have shown that early initiation of ganciclovir/valganciclovir treatment in symptomatic newborns can prevent deterioration of hearing and improve neurodevelopmental outcomes [9, 10, 39].

In our cohort, two newborns were identified with cCMV at birth, through risk factor- based screening of *n* = 106 newborns. They both had prolonged hyperbilirubinemia but did not show any other sequelae at birth or during further follow-ups. Two additional cases were identified positive for cCMV in our retrospective analysis of *n* = 1889 samples. To our knowledge none of them developed any symptoms. Although, the absolute number of cases in our study is small and the possibility of missed symptomatic cases cannot be entirely excluded,, our findings, together with the current evidence, supports the approach of recently published guidelines and recommendations on diagnosis and treatment of cCMV, that recommend a targeted, risk-based screening approach rather than universal screening in low-prevalence settings [40, 41].

## Conclusions

Our retrospective, population-based analysis of cCMV infection indicates a very low disease burden in the Western Pomerania region of northern Germany. Only a small number of cases were not identified by risk factor-based screening at birth, and these cases remained without cCMV related symptoms throughout childhood. Therefore, our study does not provide any new supporting evidence for universal screening for cCMV, particularly in light of the associated uncertainties, and is in line with the current recommendations.

## Data Availability

The datasets used and/or analyzed during the current study are available from the corresponding author on reasonable request.

## Abbreviations

APGAR: Activity, pulse, grimace, appearance, and respiration
CMV: Cytomegalovirus
cCMV: Congenital infection with cytomegalovirus
CI: Confidence interval
DBS: Dried blood spot
DNA: Deoxyribonucleic acid
ENT: Department of Otolaryngology, Head and Neck Surgery
NICU: Neonatal intensive care unit
PCR: Polymerase chain reaction
SGA: Small for gestational age
SNiP: Survey of neonates in Pomerania
SNHL: Sensorineural hearing loss
